# The impact of cocaine and heroin on the placental transfer of methadone

**DOI:** 10.1186/1477-7827-7-61

**Published:** 2009-06-11

**Authors:** Antoine Malek, Cristina Obrist, Silvana Wenzinger, Ursula von Mandach

**Affiliations:** 1 Department of Obstetrics, Zurich University Hospital, Frauenklinikstr. 10, 8091 Zurich, Switzerland

## Abstract

**Background:**

Methadone is the therapeutic agent of choice for the treatment of opiate addiction in pregnancy. The co-consumption (heroin, cocaine) which may influence the effects of methadone is frequent. Therefore, the impact of cocaine and heroin on the placental transfer of methadone and the placental tissue was investigated under in vitro conditions.

**Methods:**

Placentae (n = 24) were ex-vivo perfused with medium (m) (control, n = 6), m plus methadone (n = 6), m plus methadone and cocaine (n = 6) or m plus methadone and heroin (n = 6). Placental functionality parameters like antipyrine permeability, glucose consumption, lactate production, hormone production (hCG and leptin), microparticles release and the expression of P-glycoprotein were analysed.

**Results:**

Methadone accumulated in placental tissue. Methadone alone decreased the transfer of antipyrine from 0.60 +/- 0.07 to 0.50 +/- 0.06 (fetal/maternal ratio, mean +/- SD, P < 0.01), whereas the combination with cocaine or heroin increased it (0.56 +/- 0.08 to 0.68 +/- 0.13, P = 0.03 and 0.58 +/- 0.21 to 0.71 +/- 0.24; P = 0.18). Microparticles (MPs) released from syncytiotrophoblast into maternal circuit increased by 30% after cocaine or heroin (P < 0.05) and the expression of P-glycoprotein in the tissue increased by ≥ 49% after any drug (P < 0.05). All other measured parameters did not show any significant effect when methadone was combined with cocaine or heroine.

**Conclusion:**

The combination of cocaine or heroin with methadone increase antipyrine permeability. Changes of MPs resemble findings seen in oxidative stress of syncytiotrophoblast.

## Background

In developed countries an increase in substance abuse in pregnancy can be observed. Opiates cross the placenta easily and lead to intrauterine growth restriction (IUGR), preterm birth and spontaneous abortion [1-4]. Abstinence cannot be achieved in most of the patients and methadone is the recommended standard of care for pregnant opioid-dependent women [5]. The positive effects of methadone are an increase in birth weight and the prolongation of gestation [4,6]. In addition, women in a maintenance therapy program receive more prenatal care which improves the situation for both, mother and fetus. However, the main disadvantage of this therapy is the neonatal abstinence syndrome (NAS) which occurs in 60–80% of the newborns from mothers who consumed methadone and is more intensive than in babies who were prenatally exposed to heroin [3,7]. NAS due to opiate withdrawal may result in disruption of the mother-infant relationship, sleep-wake abnormalities, feeding difficulties, weight loss and seizures. It's unclear whether the maternal methadone dose correlates with the intensity and duration of NAS [5,8]. Nevertheless, transfer rates of methadone are higher from the fetal to the maternal circuit than vice versa [9]. This phenomenon is based on the fact that methadone is a substrate of the ATP-dependent efflux transporter protein, the P-glycoprotein (P-gp) which is expressed in the brush-border membranes of the maternal derived syncytiotrophoblast and works against the concentration gradient [7,9,10]. Because co-consumption of methadone with other drugs such as cocaine and heroin is frequent, additional drugs may influence the placental transfer of methadone and other substances by different mechanisms. In case of inhibition of the P-gp function by other drugs, the placental barrier may disrupt, and P-gp substrates may increasingly transfer to fetal circulation [11,12]. However, there exist no information whether heroin or cocaine are substrates of P-gp. Another mechanism is that of apoptosis or necrosis of the syncytiotrophoblast caused by some drugs. The syncytiotrophoblast is relatively thick in early pregnancy. At first trimester there is less syncytiotrophoblast and mostly of the trophoblast is cytotrophoblast. With increasing gestational age there is a differentiation of cytotrophoblast to syncytiotrophoblast. The release of microparticles shed from the syncytiotrophoblast into the maternal blood is generated during apoptosis or necrosis. MPs are larger than 100 nm in diameter and originate from blebbing membranes of either activated cells or cells undergoing apoptosis and mainly consist of nuclear proteins as well as nucleic acids [13]. The efficiency of transfer may be reduced by thickening of the basement membranes of capillary endothelium, obliteration of maternal vessels and the increase of the fibrinoid deposits [14-16]. Both mechanisms may affect the incidence and intensity of NAS. Cocaine as opiates leads to spontaneous abortion, low birth weight, fetal growth restriction and in addition to impaired neurodevelopment [17,18]. Because of its vasoactivity, cocaine affects the fetal vasculature and impairs placental permeability [19]. Heroin may be involved in leptin metabolism. Leptin is produced in the placenta [20,21] and regulates fetal growth and angiogenesis. In pregnancies with heroin abuse [22] as well as with IUGR [23], umbilical cord blood levels of leptin are reduced. It can therefore be assumed that heroin abuse in pregnancy may reduce placental leptin synthesis and contributes to IUGR by reducing fetal growth. Similar effects of methadone or cocaine on leptin are still unknown.

The aim of this study was therefore to investigate the placental transfer of methadone without and with addition of cocaine or heroin in the ex-vivo placenta perfusion model. Thereby placental functions as well as the response of the placental tissue on methadone were key issues.

## Methods

### Placenta collection

Intact placentae were consecutively obtained from uncomplicated term pregnancies after caesarean section at our Department. All women provided written informed consent for the study which was approved by the local institutional review board (KEK-StV-Nr. 07/07).

### Placenta perfusion

**Figure 1:**
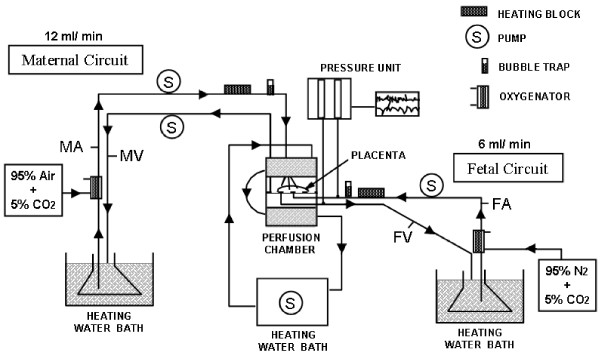
**Schematic illustration of the human placenta perfusion model (FA: fetal artery; FV: fetal vein; MA: maternal artery; MV: maternal vein)**.

Placentae were used for perfusion within 20–30 minutes after delivery. For the dual ex-vivo perfusion (earlier so called as "in-vitro" perfusion) of an isolated cotyledon the method originally described 1972 by Schneider, Panigel and Dancis [24] with a closed perfusion system for both maternal and fetal circuits was used [25]. The perfusion medium was composed of NCTC-135 tissue culture medium (ICN Biomedicals, Inc., Irvine, California, USA) diluted with Earle's buffer (1:1) with the addition of glucose (1.3 g/l; close to a physiological level), dextran 40 (10 g/l), sodium bicarbonate (2.2 g/l) bovine serum albumin (4 per cent), heparin (2500 IU/l) and clamoxyl (250 mg/l). Initial volume used on both sides (maternal and fetal) was 120–130 ml. The flow rate on the maternal side and fetal side was 12 and 6 ml/min, respectively. Two oxygenators (LSI-OX) are used: 95% N_2 _plus 5% CO_2 _for the fetal circuit and 95% air plus 5% CO_2 _for the maternal perfusate respectively. The oxygen level in the perfusion medium was 130–150 mg Hg (maternal side) and 40–60 mm Hg (fetal side), respectively (Figure 1).

Experiments using a crossover system were performed according to four different protocols (Table 1) including always a 20 minute pre-phase of open perfusion of both compartments to flush the blood out of the intervillous space and the villous vascular compartment. For each protocol six experiments, (each on one placenta) were carried out, so six placentae per group and a total of 24 placentae for all experiments were used. Each experiment included two phases (I and II) of 2 hours. The crossover system was used in relation to the fact that metabolic parameters measured under control group (protocol A) differ between phase I (2 hours) and phase II (2 hours) (0.26 vs. 0.18 μmol/g/min glucose consumption; 0.22 vs. 0.18 μmol/g/min lactate production; 60 vs. 37 mU/g/min hCG accumulation; 82 vs. 66 pg/g/min leptin accumulation). The same effect was already shown by Miller RK et al., 1985 [26] and Di Santo et al., [27]. Therefore, in the following experimental groups (protocol B, C, D), each experiment included a control phase (2 hours), which was alternately applied either in the phase I or in the phase II, while the test substances were added in the remaining other experimental phase. In protocol B methadone *(Sigma Aldrich, Fluka, CH-Buchs) *at a concentration of approximately 500 ng/ml corresponding to plasma levels following a dose of 120 mg per day was added to the medium on the maternal side at the beginning of either phase I or phase II. In protocol C, cocaine *(Sigma Aldrich, Fluka, CH-Buchs) *at a concentration of 3 mg/l together with methadone 500 ng/ml and in protocol D, heroin [28] 150 ng/ml together with methadone 500 ng/ml respectively was added. The exact methadone concentration of used initial maternal medium was verified by analytical determination simultaneously with the perfusion samples. The perfusion media were replaced on both maternal and fetal sides after phase I. In all experiments 45 nCi/ml of ^14 ^[C]antipyrine *(*radioactivity: 4.7 mCi/mmol, *Sigma Aldrich, Fluka, CH-Buchs) *was added at the beginning of each phase to the maternal perfusate to measure placental permeability from the maternal to the fetal direction. Antipyrine is a common reference marker to measure passive diffusion-dependent transfer [29].

**Table 1:** Perfusion of placentae (*n *= 24) including two phases in a crossover system

**Protocol**	***n***	**Phase I (2 hours)**	**Phase II (2 hours)**
A	3 3	m + antipyrine m + antipyrine	m + antipyrine m + antipyrine
B	3 3	m + antipyrine m + antipyrine + methadone	m + antipyrine + methadone m + antipyrine
C	3 3	m + antipyrine m + antipyrine + methadone + cocaine	m + antipyrine + methadone + cocaine m + antipyrine
D	3 3	m + antipyrine m + antipyrine + methadone + heroin	m + antipyrine + methadone + heroin m + antipyrine

Protocol A: control

m: perfusion medium

### Sample collection

Perfusion media aliquots of 5 ml from both sides (fetal and maternal) were collected at each hour of the perfusion and stored at -20°C until analysis of placental methadone levels, placental metabolism, permeability and microparticles. Tissue specimens before and after perfusion were collected and snap-frozen in liquid nitrogen and stored at -80°C until analysis of methadone and P-glycoprotein.

### Analytical methods

#### Concentrations of methadone

Methadone and its primary metabolite (2-ethyliden-1, 5-dimethyl-3, 3-diphenyl-pyrrolidine = EDDP) were analyzed using High Pressure Liquid Chromatography (HPLC) and Mass Spectrometry (MS) [30]. 0.5 ml H_2_O and 500 μl 100 mM ammonium carbonate buffer (pH 9.3) were added to 1 ml of the perfusate sample and the tissue homogenate respectively and then extracted with 5 ml n-hexane/dichlormethane (4:1 ratio v/v). The organic extract was dried completely with a rotavap (35°C) and aired additionally under nitrogen (N_2_). The residue was dissolved in 100 μl acetonitrile buffer and 300 μl ammonium acetate buffer. Aliquots were analysed by HPLC and MS. The calibration curve ranged from 0.69 to 828 ng/ml.

#### Placental metabolism

Glucose and lactate determinations were performed using an enzymatic assay of hexokinase/glucose-6-phosphate dehydrogenase and lactate dehydrogenase/glutamate pyruvate transaminase respectively [31]. Glucose consumption was calculated from the differences between initial and final levels in the medium on both maternal and fetal sides and were normalized to wet tissue weight and length of perfusion period as previously described by Di Santo et al., 2003 [27]. For the measurement of hCG and leptin in the medium, standard enzyme-linked-immunosorbent assays (ELISA) developed earlier by Malek et al. [21] were used whereas the rate of accumulation was calculated as previously described [32]. Thereby the respective protein in samples is bounded by-purified anti-hCG or anti-leptin antibodies (capture antibodies) and immobilized on plastic plates (NUNC Maxisorpy™ microplates, Nalge Nunc International, Denmark). The captured protein was detected by a second enzyme-conjugated antibody. Following the addition of a chromogenic substrate, the level of the generated colored product is measured spectrophotometrically and the protein concentration is determined from the respective standard curve.

#### Antipyrine transfer

The transfer rate of antipyrine was determined according to the ^14^C-labeled antipyrine reference method of Challier, 1985 [29].

#### Microparticles

Isolation of microparticles (MPs) was carried out with 10 ml of maternal final perfused medium and centrifugation at 3'200 × g. The supernatant was centrifuged at 10'000 × g and the pellet was re-suspended in 100 μl PBS [33-35]. The protein content was determined using the BCA™ Protein Assay Kit (*Sigma-Aldrich, Buchs, Switzerland*) with bovine serum albumin as a standard. All steps were performed at 4°C [10,16]. Identification of MPs as syncytiotrophoblastic shedding was performed by flow cytometric analysis with mouse monoclonal anti-Placental Alkaline Phosphatase (PLAP) described by Di Santo et al. 2007 [31].

#### P-glycoprotein

Isolation of brush-border membranes: 3 g tissue (mixed from three different locations of the placenta collected before and after perfusion respectively) were dissected and washed four times with PBS (83 mM NaCl, 22 mM Na2HPO4, 5.5 mM KH2PO4, pH 7.4). The tissue was homogenized for 1 minute and the tissue suspension was centrifuged at 3'200 × g for 10 min and the supernatant was further centrifuged at 10'000 × g for 30 min. Thereafter, the supernatant (1.0 ml) was centrifuged again at 100'000 × g (*Sorvall Ultraspeed Centrifuge Rotor Log, Thermo Fisher Scientific) *and the pellet was resolved in 100 μl PBS and used for western blot. Its protein content was determined using the BCA™ Protein Assay Kit (*Sigma-Aldrich, Buchs, Switzerland*) with bovine serum albumin as a standard. All steps were performed at 4°C [9,20].

Western blot: The identification of P-glycoprotein expression was achieved using 10.0% SDS/polyacrylamide gel electrophoresis. The amount of sample protein loaded on each well was 40 μg. The procedure follows the method of the trouble shooting protocol of the R&D System [36]. The transfer of the protein on the nitrocellulose membrane (*ECL Hybond, nitrocellulose membrane, Amersham Biosciences, Buckinghamshire*) was performed using the tank blotting system overnight. Non-specific binding domains were blocked by incubation with 5% non-fat dry milk in TBS for 1 h. The blots were incubated with the primary murine monoclonal anti-P-glycoprotein antibody (diluted 1:200) (*Sigma-Aldrich*) and the anti-beta-actin antibody (diluted 1:500) (*Sigma-Aldrich*) as an internal standard for one hour. The membrane was washed and incubated with the goat antimouse horseradish peroxidase-conjugated antibodiy (diluted 1:1000) (*Dako*) as secondary antibody for one hour. The detection was carried out using chemiluminescence and the visualization was performed at chemiluminescence films (*ECC hyper film, high performance chemiluminescence film, Amersham Biosciences, Buckinghamshire, UK*) [9,20]. The density of the simultaneously obtained bands of P-gp and beta-actin was measured using a densiometer. Values of P-gp were normalized to the respective value of the beta-actin in the same run.

#### Calculations

The glucose consumption and lactate production were calculated from the differences between initial and final levels in the perfusate on both, the maternal and fetal side, and normalized to tissue weight and length of perfusion period.

The net production (NP) of hCG and leptin after 4 h was calculated with the formula NP = A_tot _+ T_E _- T_0_, where A_tot _means the hormone accumulation in the perfusate, T_E _the tissue content determined after perfusion and T_0 _the tissue content before initiation of the experiment. Results were normalized to tissue weight and to T_0_.

#### Statistics

Statistical analysis was performed using Prism version 3.0 *(GraphPad Software, San Diego California, USA)*. Mean ± SD were calculated. The Mann-Whitney U-test was used to estimate the statistical significance of the differences between or in experimental groups respectively. Differences were considered significant if *P *< 0.05.

## Results

### Methadone concentration

In the perfusion with methadone alone, a concentration of 389 ± 79 ng/ml was applied either in the first or in the second phase (Table 2). After 2 hours the methadone concentration in the perfusate from the maternal side as well as from the fetal side was below the detection limit of < 0.69 ng/ml. In perfusions with methadone plus cocaine 3 mg/l, the methadone uptake was slower, which was indicated by the detection of methadone (1.6 ± 1.1 ng/ml) on the maternal side, while on the fetal side the level remained again undetectable. When heroin was added, the methadone uptake was more reduced than with cocaine and the concentrations were measurable both, on the fetal and on the maternal side with a fetal/maternal ratio of 1:7. In all experiments methadone increased in the tissue (cotyledon), whereas the highest accumulation was seen in perfusions with methadone plus heroin (172% vs. methadone alone, *P *= 0.008). In cases where methadone was applied in the first phase of the perfusion, no methadone could be detected in the perfusates of the second phase indicating that there was no wash out of methadone from the tissue. Concentrations of the metabolite were under the detection limit in all samples.

**Table 2:** Methadone concentration measured in the initial maternal medium (M_0_) and after two hours of perfusion in the maternal medium (M_E_), the fetal medium (F_E_) and in the perfused tissue (T_E_)

	**M_E _[ng/ml]** **(% Recovery)**	**F_E _[ng/ml]** **(% Recovery)**	**T_E _[ng/g]** **(% Recovery)**	**Cotyledon** **Weight [g]**
Perfusion with methadone (*n *= 6)	<0.69 (0)	<0.69 (0)	1987 ± 587 (94)	20.1 ± 3.4
Perfusion with methadone plus cocaine (*n *= 6)	1.6 ± 1.1 (0.6)	<0.69 (0)	2814 ± 352 (96)	19.6 ± 2.3
Perfusion with methadone plus heroin (*n *= 6)	57 ± 22 (6)	8 ± 10 (1)	3427 ± 374^a^(92)	20.3 ± 2.7

The detection limit of methadone was 0.69 ng/ml.

Values are mean ± SD

^a^*P *< 0.01 vs. perfusion with methadone

### Placental metabolism

There was no significant difference in the glucose consumption (μmol/g/min) and lactate production (μmol/g/min) between phase I (0.16 ± 0.04 glucose; 0.19 ± 0.04 lactate) and phase II (0.14 ± 0.03 glucose; 0.17 ± 0.04 lactate) in any group of perfusion. There was also no significant difference between perfusions with and without drugs (controls).

The tissue content for hCG and leptin was significantly higher at the end than before the perfusion in all experiments excluding that with methadone/heroin. In case of methadone/heroin addition the tissue content for hCG before vs. after perfusion was unchanged whereas the leptin content decreased significantly after perfusion (*P *< 0.05). The net production of both hormones when related to the tissue initial content gave values over 100% in every group (Table 3).

**Table 3:** Tissue content before perfusion (T_0_) and after 4 hours of perfusion (T_E_), accumulation (A_tot_) and net production (NP) as well as percent NP of T_0 _(% NP) of hCG and leptin

		**T**_0_	**T**_E_	**A**_tot_	**NP**	**% NP**
Control perfusion (*n *= 6)	hCG [mU/g]	6100 ± 4538	9028 ± 7205^b^	11608 ± 7179	14536 ± 8956	299 ± 80
	Leptin [pg/g]	7944 ± 5189	10050 ± 7672^c^	18122 ± 6824	20228 ± 8752	320 ± 136
Perfusion with methadone (*n *= 6)	hCG [mU/g]	3422 ± 2357	3996 ± 1908^b^	6711 ± 3318	7285 ± 2868	269 ± 59
	Leptin [pg/g]	3673 ± 643	5590 ± 3431^a^	14380 ± 4023	16298 ± 6737	480 ± 257
Perfusion with methadone plus cocaine (*n *= 6)	hCG [mU/g]	4530 ± 2202	5305 ± 4429^b^	10539 ± 6563	11313 ± 8219	224 ± 116
	Leptin [pg/g]	2920 ± 544	4370 ± 2303^b^	37744 ± 20897	39195 ± 22796	1200 ± 573
Perfusion with methadone plus heroin (*n *= 6)	hCG [mU/g]	3852 ± 1649	4336 ± 2088	5176 ± 2067	5660 ± 2647	169 ± 81
	Leptin [pg/g]	9856 ± 6146	5568 ± 2194^a^	17949 ± 9578	13662 ± 7026	191 ± 573

Values are mean ± SD

^a ^P < 0.05 after vs. before; ^b ^P < 0.01 after vs. before; ^c ^P < 0.001 after vs. before.

There is no statistical difference in NP of hCG or leptin between control perfusion and each of the other experimental groups.

### Transfer of antipyrine

Antipyrine transfer profiles from maternal to fetal side were similar in phase 1 and phase 2 (control experiments).

**Figure 2:**
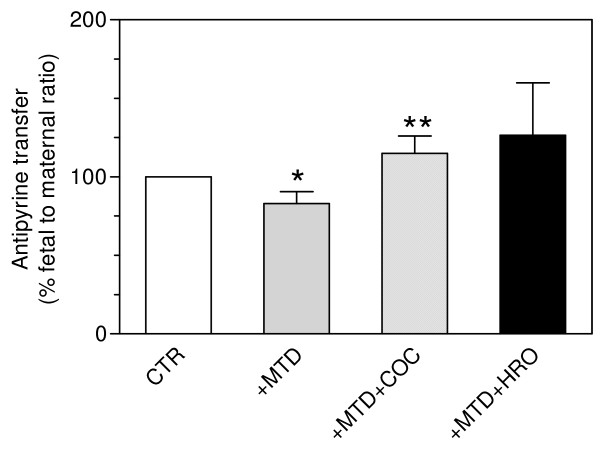
**Effect of methadone (+MTD), methadone plus cocaine (+MTD+COC) and methadone plus heroin (+MTD+HRO) on antipyrine transfer**. Values are mean ± SD (*n *= 6) and expressed as percentage of control (CTR = 100%). *P < 0.01 vs. CTR, **P = 0.03 vs. CTR.

Methadone decreased the transfer of antipyrine. The mean fetal to maternal ratio was 80% (0.50 ± 0.06) of the control ratio (0.60 ± 0.07, *P *< 0.01). The perfusion with methadone plus cocaine increased the transfer of antipyrine; the fetal to maternal ratio was 120% (0.68 ± 0.13) of the control ratio (0.56 ± 0.08, *P *= 0.03). A non-significant increase in the transfer was seen when methadone plus heroin was perfused indicating a fetal to maternal ratio of 0.71 ± 0.24, which was 127% of the control ratio (0.58 ± 0.21, *P = *0.179) (Figure 2).

### Microparticles

**Figure 3:**
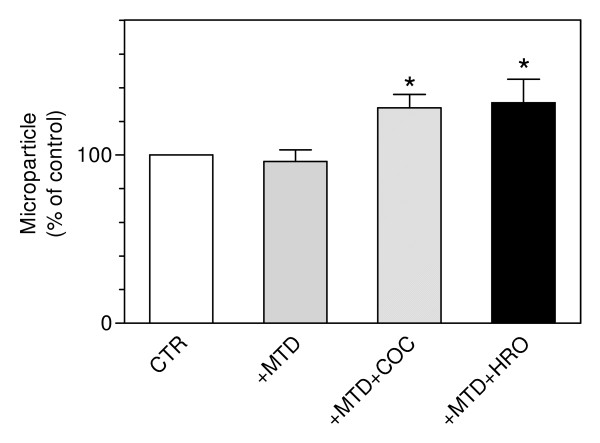
**Effect of methadone (+MTD), methadone plus cocaine (+MTD+COC) and methadone plus heroin (+MTD+HRO) respectively on the release of microparticles into the maternal circuit quantified by total protein content**. Values are expressed as mean ± SD (*n *= 6) of the percentage of the control (CTR = 100%). *P = 0.03 vs. CTR

The concentration of the released microparticles (MPs) isolated from the maternal side after perfusion without addition of any drug (control) was 2190 ± 655 ng protein/g/min. A non-significant reduction in the release of MPs (96 ± 7% of the the control) was observed when methadone was added. The accumulation of MPs increased after perfusion with methadone/cocaine 128 ± 8% of the control (*P *= 0.03). In case of the perfusion with methadone/heroin the MP's percentage increased to 134 ± 14 of the control (*P *= 0.03). No significant difference to the control was seen after the perfusion of methadone alone (Figure 3). The percentage of trophoblast-derived MPs as indicated by PLAP was unchanged in all experimental groups.

### P-glycoprotein

**Figure 4:**
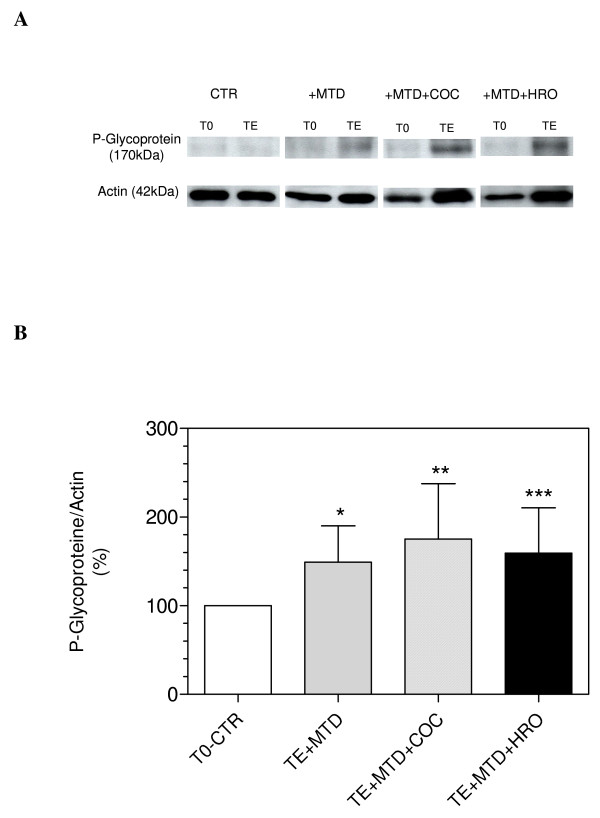
**A. Western blot analysis of P-glycoprotein expression in the isolated brush border membrane before perfusion (T0) and after perfusion (TE). CTR: control; +MTD: methadone; +MTD+COC: methadone plus cocaine; +MTD+HRO: methadone plus heroin. B. P-glycoprotein expression (percentage of actin) in the isolated brush border membrane after perfusion with methadone (TE+MTD), methadone plus cocaine (TE+MTD+COC) and methadone plus heroin (TE+MTD+HRO).** Values are expressed as mean ± SD (*n *= 6) of the percentage of the control (tissue before perfusion: T0-CTR = 100%). *P = 0.03 vs. T0-CTR, **P = 0.01 vs. T0-CTR, ***P = 0.03 vs. T0-CTR.

The P-glycoprotein expression (percentage of actin expression) in the tissue increased significantly during the perfusion with methadone alone or methadone in combination with cocaine or heroin by 49 ± 41% (*P *= 0.03), 75 ± 63% (*P *= 0.01) and 59 ± 51% (*P *= 0.03) respectively compared to the tissue before perfusion (control) (Figure 4A, B).

## Discussion

Transplacental transfer of drugs (or other substances) and the amount that enters the fetal circulation is determined by at least three processes: simple diffusion, efflux transporters, and biotransformation by metabolic enzymes. The syncytiotrophoblast layer with the brush border membrane contains several transporter proteins and is in direct contact with the maternal blood in the intervillous space. It's therefore extremely sensitive for changes induced by maternal uptake. In the present study we found differing mechanisms of placental function and tissue reaction for methadone perfused in a closed circulation: There was no measurable concentration of methadone in the fetal circulation when methadone was perfused alone or with the addition of cocaine or heroin, respectively. Of course this could be proven in an open system where the re-uptake of transferred substances (methadone and heroin) is not possible. However, these results are in contrast to that of other authors [7,10], who perfused methadone alone. Nanovskaya et al. demonstrated a transfer into the fetal circuit of 31% [10] and Nekhayeva et al. [7] detected methadone in the fetal circuit already within 5 minutes of its addition to the maternal reservoir even though they used a lower concentration of methadone than we did (100–400 ng/ml vs. 500 ng/ml) used. One reason for this difference may be the use of different methods. While in the study of Nekhayeva IA et al. [7] the perfusion lasted for 4 hours and methadone was measured by the radioactivity of the ^3^H-labelled methadone, we perfused methadone only for 2 hours and measured its concentration by HPLC and MS.

Our control experiments showed similar values for placental metabolism and function (glucose consumption, lactate production, production and accumulation of hCG and leptin) as found in previous studies with the same ex-vivo placenta perfusion model [21,30-32]. These results demonstrate that the perfused tissue maintains under in vitro condition the ability for de-novo synthesis of hCG and leptin. The addition of cocaine or heroin to methadone showed no different results of glucose consumption, lactate production, hCG accumulation and production. There was only a nonsignificant difference in the leptin production in the experiments with heroin. As it was already observed under in vivo conditions where serum leptin levels decreased in patients with heroin addiction [22], the consequences of modified leptin production during heroin consumption in pregnancy should be verified in further experiments. Consequently, until now, it has to be postulated that methadone plus cocaine or methadone plus heroin do not affect placental viability. In case of cocaine the present results do not agree with those reported in a previous study [19,37], where cocaine (without methadone) led to a significant decrease in the hCG release to the maternal circuit.

In contrast to Nekhayeva et al., we could show that methadone has a significant influence on placental permeability. In the perfusion with methadone alone, the antipyrine transfer decreased. This fact could lead to a dysfunction in the supply of the fetus with oxygen and nutrients from the maternal circulation. Intrauterine growth restriction observed under in vivo supplementation with methadone [1] could be based on this mechanism. In contrast to the experiments with methadone alone antipyrine transfer increased significantly in the phases where cocaine or heroin was added. Even though the supply of the fetus with oxygen and nutrients may be better, the amelioration of the placental permeability could affect the barrier function of the placenta adversely. Consequently, more toxic substances or bacteria and viruses may cross the placenta and harm the fetus. Previous studies reported increased prevalence of infectious diagnoses in cocaine-exposed infants [38].

Cocaine and heroin increased the MPs production into the maternal circuit. Previous reports have shown that under different in vitro conditions the production of MPs can differ [16,39,40]. Under low oxygen (2%) there was an increased proliferation of the cytotrophoblast and lack of the fusion with syncytiotrophoblast, so shedding of MPs was predominantly the result of necrosis [39]. In this study we have used the already validated method of the ex-vivo placenta perfusion using 95% air on the maternal circuit [33]. It was also shown that, this method is a good model to investigate the shedding of MPs from synyctiotrophoblast as a sign for oxidative stress seen in preeclampsia [33]. One possibility that we can postulate, is that methadone alone does not influence the tissue structure whereas the combination with cocaine and heroin may induce oxidative stress. Further work is required to confirm this and the mechanisms involved.

Methadone is a substrate of the ATP-dependent efflux transporter P-gp [9-11,41]. Our results showed increasing expression after perfusion with methadone but no additional increase if cocaine or heroin were added to methadone. As it was shown by other authors P-gp expression is 30% higher in preterm than in term placentas [10]. Increased activity of P-gp may therefore affect the incidence and intensity of NAS in babies of women who were treated with methadone during pregnancy and who delivered preterm in most cases.

## Conclusion

As the consumption of illegal drugs, especially cocaine is increasing in many countries as demonstrated by the World Drug Report 2007 [42], our results concerning cocaine and heroin causing an increased antipyrine transfer and possibly inducing oxidative stress in placental tissue, although we have not measured it directly, may improve the practical management in monitoring pregnant women. It should clarify the efficiency of a consequent and exclusive maintenance therapy with methadone during pregnancy.

## Competing interests

The authors declare that they have no competing interests.

## Authors' contributions

AM and UVM had participated in the design of the study. The experiments were carried out by AM, CO and SW. CO and SW had helped in collection of samples, measurement of functionality parameters and data analysis. AM and UVM drafted and wrote the manuscript. All authors have read and approved the final manuscript.
